# Cell-Free Glycoengineering of the Recombinant SARS-CoV-2 Spike Glycoprotein

**DOI:** 10.3389/fbioe.2021.699025

**Published:** 2021-08-16

**Authors:** Johannes Ruhnau, Valerian Grote, Mariana Juarez-Osorio, Dunja Bruder, Reza Mahour, Erdmann Rapp, Thomas F. T. Rexer, Udo Reichl

**Affiliations:** ^1^ Max Planck Institute for Dynamics of Complex Technical Systems, Bioprocess Engineering, Magdeburg, Germany; ^2^ Infection Immunology, Institute of Medical Microbiology, Infection Prevention and Control, Health Campus Immunology, Infectiology and Inflammation, Otto-von-Guericke University Magdeburg, Magdeburg, Germany; ^3^ Immune Regulation Group, Helmholtz Centre for Infection Research, Braunschweig, Germany; ^4^ glyXera GmbH, Magdeburg, Germany; ^5^ Otto-von-Guericke University Magdeburg, Chair of Bioprocess Engineering, Magdeburg, Germany

**Keywords:** SARS-CoV-2, COVID- 19, glycoengineering, subunit vaccine, cell-free synthetic biology

## Abstract

The baculovirus-insect cell expression system is readily utilized to produce viral glycoproteins for research as well as for subunit vaccines and vaccine candidates, for instance against SARS-CoV-2 infections. However, the glycoforms of recombinant proteins derived from this expression system are inherently different from mammalian cell-derived glycoforms with mainly complex-type *N-*glycans attached, and the impact of these differences in protein glycosylation on the immunogenicity is severely under investigated. This applies also to the SARS-CoV-2 spike glycoprotein, which is the antigen target of all licensed vaccines and vaccine candidates including virus like particles and subunit vaccines that are variants of the spike protein. Here, we expressed the transmembrane-deleted human *β*-1,2 N-acetlyglucosamintransferases I and II (MGAT1ΔTM and MGAT2ΔTM) and the *β*-1,4-galactosyltransferase (GalTΔTM) in *E. coli* to *in-vitro* remodel the *N*-glycans of a recombinant SARS-CoV-2 spike glycoprotein derived from insect cells. In a cell-free sequential one-pot reaction, fucosylated and afucosylated paucimannose-type *N*-glycans were converted to complex-type galactosylated *N*-glycans. In the future, this *in-vitro* glycoengineering approach can be used to efficiently generate a wide range of *N*-glycans on antigens considered as vaccine candidates for animal trials and preclinical testing to better characterize the impact of *N*-glycosylation on immunity and to improve the efficacy of protein subunit vaccines.

## Introduction

Most epidemics caused by viral infections that are associated with a significant death toll were caused by enveloped viruses such as influenza A virus, human immunodeficiency virus (HIV), Zika virus, Yellow fever virus, Dengue virus and Ebolavirus. Often, the main target for neutralizing antibodies to evoke a strong immune response is a glycosylated envelope membrane protein. Thus, in the development of vaccines, glycoproteins are typically in the focus of interest. In general, the glycosylation of proteins plays a critical role regarding structure, function, solubility, stability, trafficking, and ligand-binding (Imperiali and O’connor, 1999; Dalziel et al., 2014; Varki, 2017). Furthermore, glycosylation plays a major role for pharmacokinetics and pharmacodynamics of biologics and for pathogen-host interaction (Bagdonaite and Wandall, 2018; Cymer et al., 2018; Watanabe et al., 2019). In viral pathogenesis, glycosylation affects the attachment and release of virus particles as well as immune evasion (Bagdonaite and Wandall, 2018; Watanabe et al., 2019; Schön et al., 2020). Especially the latter is a major hurdle for vaccine design. The mode of actions that are known to be employed to invade the immune system are secretion and shedding of glycoproteins that function as a decoy to the immune system, and the shielding of epitopes (Watanabe et al., 2019). The latter is facilitated by occluding antigenic epitopes with host-derived glycans that are obtained through hijacking the host’s cellular glycosylation machinery (Schwarzer et al., 2009; Francica et al., 2010; Helle et al., 2011; Rödig et al., 2011; Rödig et al., 2013; Sommerstein et al., 2015; Behrens et al., 2016; Gram et al., 2016; Walls et al., 2016; Pralow et al., 2021). Moreover, it has been shown that also the glycoform itself can have an impact on binding and transmission assay as well as on transmissibility, antigenicity, and immunogenicity in animal models (Lin et al., 2003; Hütter et al., 2013; Chen et al., 2014; Li et al., 2016; Go et al., 2017). While it is assumed that immunogenic antigens benefit from mimicking the glycosylation of host cell proteins, it has also been proposed that modification of specific terminal sugar residues could be used to amplify vaccine efficacy (Galili, 2020; Chen, 2021). However, due to the complexity of protein glycosylation and the prevailing lack of methods to introduce defined modifications in the glycan composition of the proteins of interest, the topic is under investigated (Watanabe et al., 2019; Grant et al., 2020; Schön et al., 2020).

The ongoing corona virus disease 2019 (COVID-19) pandemic is caused by the severe acute respiratory syndrome coronavirus 2 (SARS-CoV-2)—a single-stranded, positive-sense RNA virus (Walls et al., 2020). Its membrane envelope consists of three membrane proteins: the surface spike (S) glycoprotein, an integral membrane protein and an envelope protein (Wan et al., 2020; Zhou et al., 2020). Virus entry into human host cells is mediated by the S glycoprotein that binds to angiotensin-converting enzyme 2 (Walls et al., 2020). The S protein has 22 N-linked glycosylation sites. Thus, it is significantly more glycosylated than, for instance, the influenza A hemagglutinin (Wrapp et al., 2020). For the SARS-CoV-1 spike protein it has been shown previously that *N*-glycans significantly impact antibody response and neutralizing antibody levels (Chen et al., 2014; Walls et al., 2020).

For the investigation of the impact of glycoforms on the immunogenicity, mainly animal cell lines such as HEK and CHO cells that produce differentially glycosylated proteins are employed (Lin et al., 2013; Schön et al., 2020). However, due to need to develop specific expression protocols for each cell line, this approach is highly work-intensive. Additionally, the inherent macro- and microheterogeneity of glycoproteins complicate the elucidation of the role of specific glycans in, for instance, regarding their immunogenicity in animal models.

Over the past years the establishment of protocols for expression of eukaryotic and bacterial glycosyltransferases has facilitated the processing of glycans in cell-free one-pot reactions. As a platform technology, the corresponding *in-vitro* glycoengineering approaches have the potential to tailor the glycoform of proteins independent of the expression systems used (Van Landuyt et al., 2019; Rexer et al., 2020a). In our study, recombinant human *β*-1,2 N-acetlyglucosamintransferases I and II (MGAT1ΔTM and MGAT2 ΔTM) and *β*-1,4-galactosyltransferase (GalTΔTM) expressed in *E. coli* were utilized to convert insect cell-derived paucimannose structures of recombinant SARS-CoV-2 spike glycoprotein to typical mammalian, complex-type galactosylated structures in a cell-free one-pot reaction (Fujiyama et al., 2001; Boeggeman et al., 2003; Bendiak, 2014; Ramakrishnan and Qasba, 2014; Stanley, 2014). Glycan structures were analyzed using multiplexed capillary gel electrophoresis with laser-induced fluorescence detection (xCGE-LIF) and Matrix-assisted laser desorption/ionization time-of-flight mass spectrometry (MALDI-TOF-MS). Results obtained clearly demonstrate that a large fraction of fucosylated and afucosylated, Man3-glycans were transferred to biantennary G2 and G2F structures (also Table 1).

**TABLE 1:** N-glycan categories and nomenclature for all detected and referenced structures with the exception of oligomannose-type N-glycans. The monosaccharide building blocks are mannose (green circle), GlcNAc (blue square), fucose (red triangle) and galactose (yellow circle).

**Paucimannose-type**	Man2F	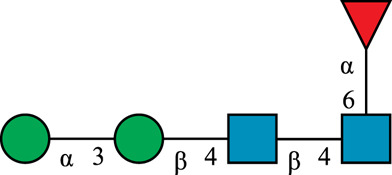		
		Man3	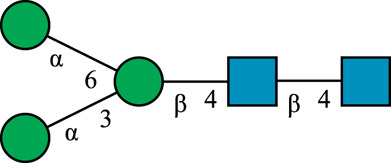	Man3F	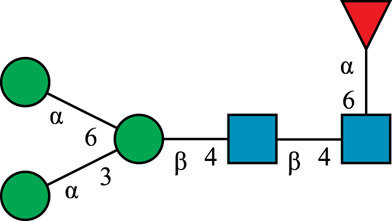
**Hybrid-type**	G0-Gn (3)	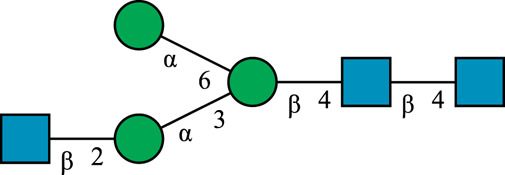	G0F-Gn (3)	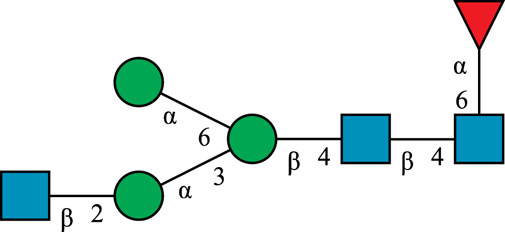
		G1F-Gn (3)	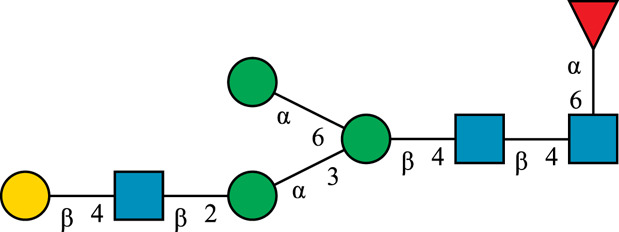		
**Complex-type**	G0	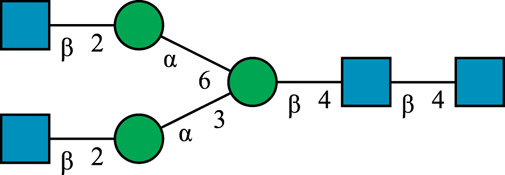	G0F	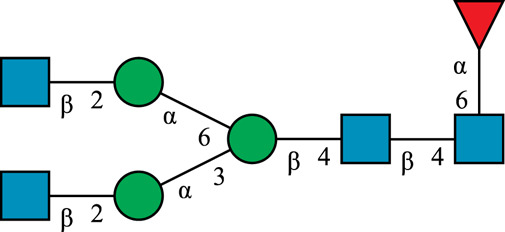
		G2	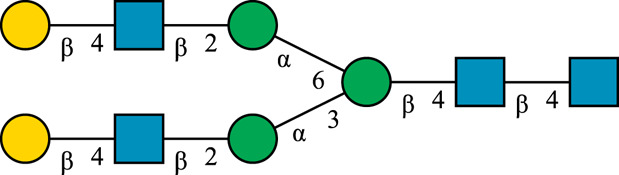	G2F	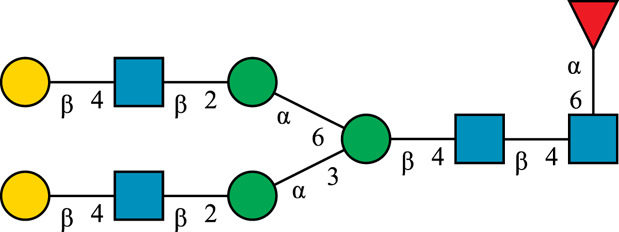

## Materials and Methods

### Enzymes

SARS-CoV-2 spike protein containing the S1 subunit and the S2 subunit ectodomain was purchased from SinoBiologica (Beijing, PR China). The recombinant protein was produced using the baculovirus-insect-cell expression system using High-Five™ cells. The protein bears a C-terminal His-tag. For all other materials see supporting information (SI).

#### Gene Expression

Genes encoding for the trans-membrane deleted (ΔTM) variants of *Homo sapiens α*-1,3-mannosyl-glycoprotein 2-*β*-N-acetylglucosaminyltransferase (MGAT1ΔTM) (E.C. 2.4.1.201), *α*-1,6-mannosyl-glycoprotein 2-*β*-N-acetylglucosaminyltransferase (MGAT2ΔTM) (E.C. 2.4.1.143) and *β*-N-acetylglucosaminylglycopeptide *β*-1,4-galactosyltransferase (GalTΔTM) (E.C. 2.4.1.38) were expressed in *E. coli*. All constructs are bearing a 6 x histidine-tag (His-tag). For information on the cultivation, strains and vectors used Supplementary Material.

#### Purification by Ion Metal Affinity Chromatography

*E. coli* cells were lysed at 4°C by high-pressure cell disruption (3 cycles, 400–600 bar) using an HPL6 homogenizer (Maximator GmbH, Nordhausen, Germany) followed by centrifugation at 7,200 × g for 20 min at 4°C to precipitate cell debris. The overexpressed enzymes were filtered through 8 µm syringe filters and then purified by ion metal chromatography using an ÄKTA™ start system equipped with HisTrap™ HP columns (1 ml) (both GE Healthcare Life Sciences, Little Chalfont, United Kingdom). A buffer exchange was carried out to remove excess imidazole using an Amicon® Ultra-15 Centrifugal Filter Unit—3 kDa MW cutoff (UFC900308, Darmstadt, Germany) using standard procedures. Enzymes were stored in 50% (v/v) glycerol stock solutions at -20°C. Enzyme concentrations were determined by performing a bicinchoninic acid (BCA) assay using the Pierce™ BCA Protein Assay Kit (Thermo Fisher Scientific; Waltham, United States).

### One-Pot *In-Vitro* Glycoengineering Reactions

Reactions were performed by sequential addition of enzymes in buffered (25 mM HEPES, pH 6.5) aqueous solutions supplemented with 10 mM MnCl_2_ at 37°C under shaking (550 rpm). The initial reaction volume (1 ml) contained 0.1 μg/ml of SARS-CoV-2 spike protein, 4 mM UDP-GlcNAc and 0.2 μg/μL MGAT1ΔTM. After a reaction time of 12 h, 150 µL of a buffered solution containing 4 mM UDP-GlcNAc and 0.85 μg/μL MGAT2ΔTM was added to 500 µL of the reaction. After 12 more hours, 175 µL of a buffered solution containing 4 mM UDP-galactose and 0.56 μg/μL GalTΔTM was added to 325 µL of the reaction mix. Three aliquots of the reactions were taken for N-glycan analysis by xCGE-LIF before the addition of each enzyme and at the end of the reaction (12 h after GalTΔTM addition).

#### Sample Pre-treatment: PNGase F Digest of N-Glycosylated Proteins

Samples from *in-vitro* glycoengineering reactions were vacuum evaporated. At least 1 μg *N*-glycosylated protein sample was linearized and denatured by adding 2 µL 2% (w/v) SDS in PBS buffer (pH 7.2) and subsequent heating at 60°C for 10 min. Samples were cooled down to room temperature. 4 µL 8% (w/v) IGEPAL in PBS and 1 µL of a 1 U/µL PNGase F solution were added. Samples were incubated for 1 h at 37°C, vacuum evaporated and dissolved in 20 µL LC-MS grade H_2_O.

#### Multiplexed Capillary Gel Electrophoresis With Laser-Induced Fluorescence Detection Based N-Glycan Analysis

*N*-glycan analysis based on xCGE-LIF was conducted using a glyXboxCE™-system (glyXera, Magdeburg, Germany) according to (Hennig et al., 2015; Hennig et al., 2016). Briefly, 2 µL of each sample was used for fluorescent labelling of *N*-glycans with 8-aminopyrene-1,3,6-trisulfonic acid (APTS) following post derivatization clean-up by hydrophilic interaction liquid chromatography-solid phase extraction (HILIC-SPE) with the glyXprep16™ kit (glyXera). Data processing, normalization of migration times and annotation of *N*-glycan fingerprints were performed with glyXtool™ software (glyXera).

#### Matrix-Assisted Laser Desorption/Ionization Time-of-Flight Mass Spectrometry Based N-Glycan Analysis

MALDI-TOF-MS analysis of released *N*-glycans was performed as described previously (Selman et al., 2011; Fischöder et al., 2019). Briefly, 0.9 cm cotton rope was used for Cotton HILIC SPE. The stationary phase was equilibrated with 50 µL LC-MS grade H_2_O followed by 50 µL 85% ACN_aq_. 10 µL of released *N*-glycans were adjusted to 70 µL 85% ACN_aq_ with 1% TFA and loaded onto the HILIC phase. Following two washing steps with 50 µL 85% ACN_aq_ with 1% TFA and 50 µL 85% ACN_aq_, the samples were eluted in 70 µL LC-MS grade H_2_O, vacuum evaporated and dissolved in 20 µL LC-MS grade H_2_O. For the MALDI-TOF-MS analysis 0.5 µL super-dihydroxybenzoic acid (S-DHB) (≥99.0%, Sigma-Aldrich, Steinheim, Germany) matrix (10 mg/ml) in 30% (v/v) ACN_aq_, 0.1% (v/v) TFA, 2 mM NaCl was spotted onto a MTP AnchorChip 800/384 TF MALDI target (Bruker Daltonics, Bremen, Germany). Subsequently 1 µL sample was applied onto the dried matrix layer. Measurements were carried out on an ultrafleXtreme MALDI-TOF/TOF MS (Bruker Daltonics, Bremen, Germany) in reflectron positive ion mode. Data was processed with the top-hat filter and the adjacent-averaging algorithm using flexAnalysis version 3.3 Build 80 (Bruker Daltonics, Bremen, Germany).

### N-Glycan Nomenclature

*N*-Glycan nomenclature was adopted from Stanley et al. (2015). Depiction of *N*-glycan structures followed the Symbol Nomenclature for Glycans (SNFG) guidelines (Neelamegham et al., 2019). The *N*-glycan sketches in this manuscript were produced using the “Glycan Builder2” software tool (Tsuchiya et al., 2017). *N*-Glycans are typically categorized into paucimannose-, oligomannose-, hybrid- and complex-type structures.

## Results

### Pathway Design

**FIGURE 1:**
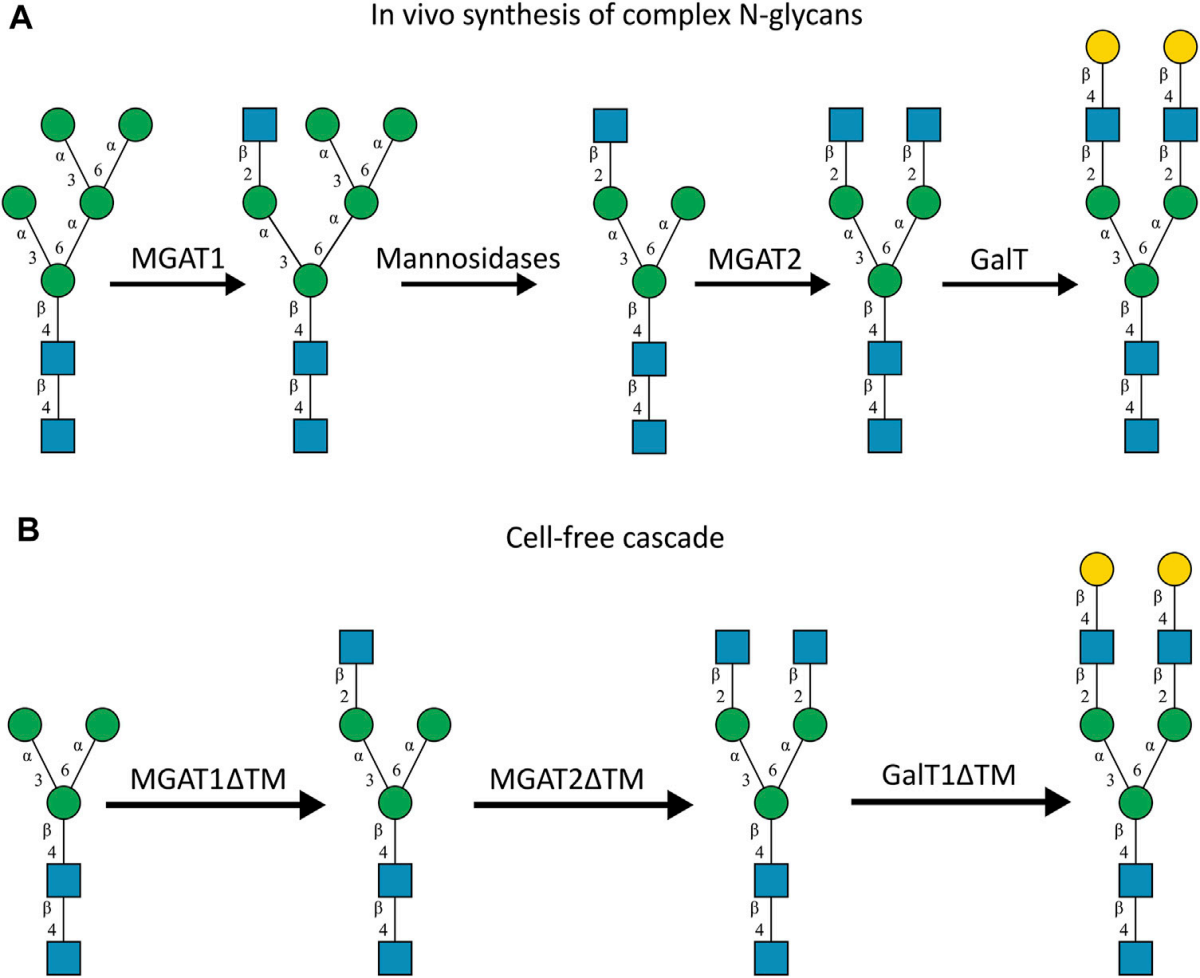
**(A)***In-vivo* the oligomannose-type *N*-glycan Man5 is converted into complex-type *N*-glycans by mannosidases and MGAT1, MGAT2 and GalT. Substrates for these reactions are UDP-GlcNAc and UDP-galactose, respectively. **(B)** This process can be remodelled *in-vitro* to synthesize complex-type structures on insect cell-derived recombinant proteins with paucimannose-type N-glycans, like Man3.

The human *in-vivo* cascade reaction for the generation of complex-type *N*-glycans from the conserved ER-derived oligomannose-type *N*-glycan precursor GlcNAc_2_Man_9_Glc_3_, was in part re-modelled *in-vitro* to generate fully galactosylated complex-type *N*-glycans starting from insect cell-derived paucimannose-type *N*-glycans. Central to the construction of the simplified *in-vitro* cascade is the ability of human MGAT1 to utilize Man3 and Man3F as substrates, which allows circumventing the application of recombinant mannosidases (Figure 1). For the production of the G2 structure from paucimannose-type *N*-glycans, the three recombinant glycosyltransferases MGAT1ΔTM, MGAT2ΔTM and GalTΔTM were successfully produced in *E. coli* (Supplementary Material). Enzyme concentrations of typically 1.3 mg/ml after ion metal affinity chromatography (IMAC) and buffer exchange were obtained. In scouting experiments, it was confirmed that all enzymes are active in the buffered solutions (pH 6.5) with MnCl_2_ supplemented as a co-factor (data not shown).

### Glycoform of the Unprocessed Recombinant SARS-CoV-2 Spike Glycoprotein

**FIGURE 2:**
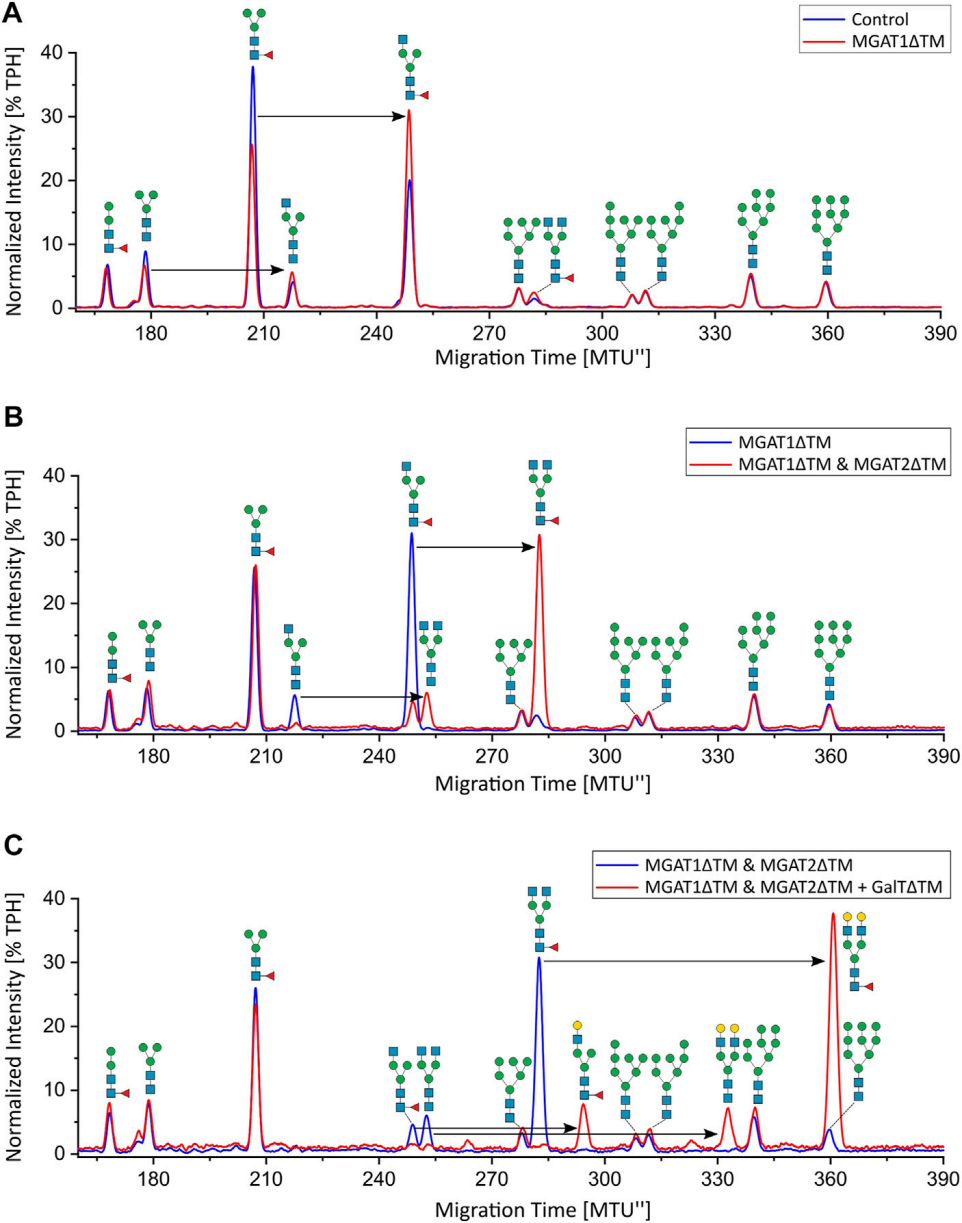
xCGE-LIF *N*-glycan fingerprints of unprocessed and *in-vitro* glycoengineered SARS-CoV-2 spike protein *N*-glycans. *N*-glycosylation pattern of the spike protein: **(A)** unprocessed (blue) and 12 h after start of the reaction with MGAT1ΔTM (red); **(B)** 12 h after start of the reaction with MGAT1ΔTM (blue) and 12 h after the addition of MGAT2ΔTM (red); **(C)** 12 h after the addition of MGAT2ΔTM (blue) and 12 h after addition of GalTΔTM (red). TPH, total peak height.

**FIGURE 3:**
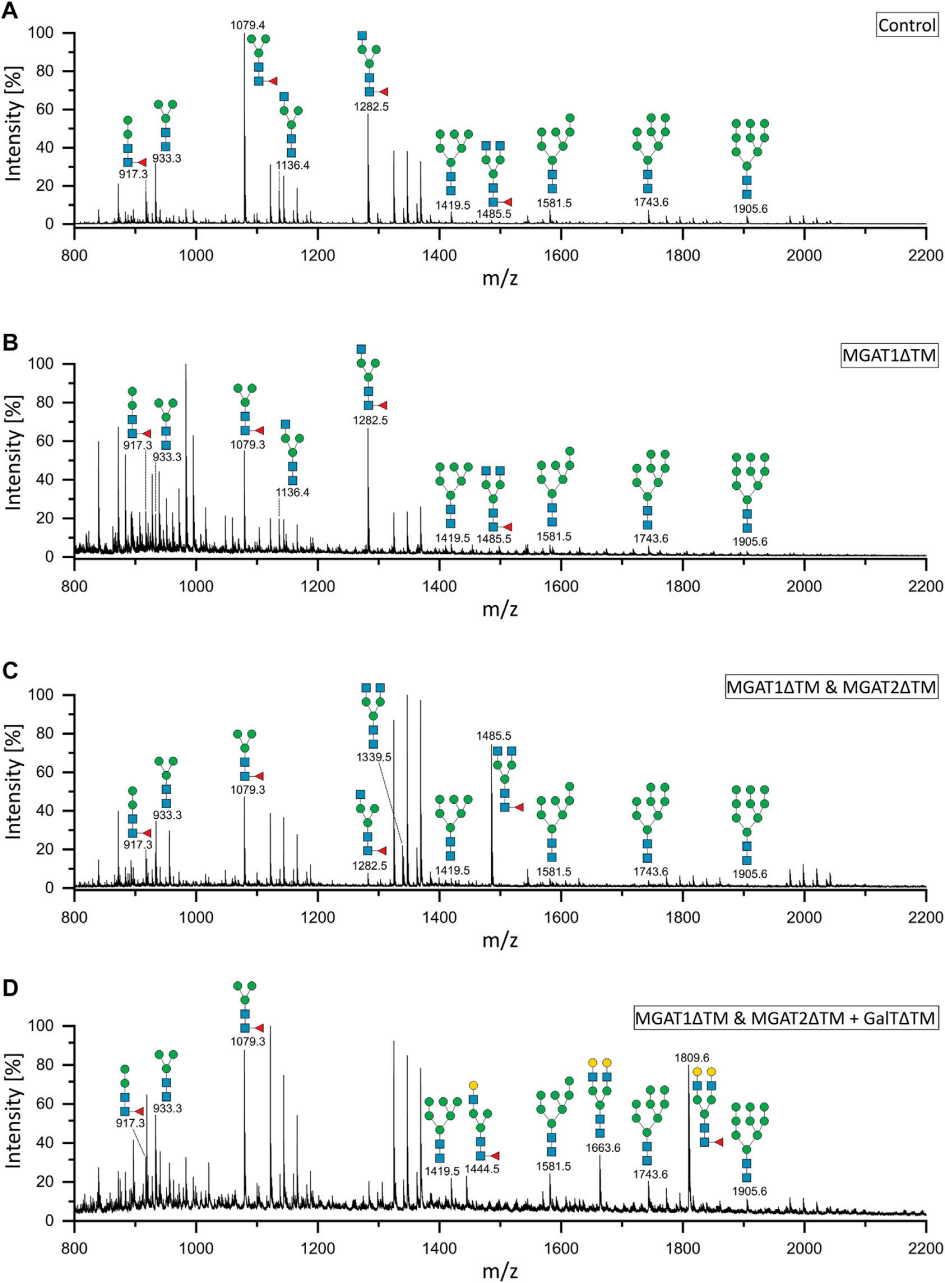
MALDI-TOF mass spectra of the unprocessed and glycoengineered SARS-CoV-2 spike protein glycoforms. *N*-glycans were detected in reflectron positive ion mode as sodium adducts [(M + Na)+]. **(A)** Unprocessed spike protein. **(B)** 12 h after start of the reaction with MGAT1ΔTM. **(C)** 12 h after the addition of MGAT2ΔTM. **(D)** 12 h after the addition of GalTΔTM. Only the peaks that depict *N*-glycans are annotated.

Analytical characterization of the unprocessed and glycoengineered SARS-CoV 2 spike protein was achieved by the two orthogonal methods xCGE-LIF and MALDI-TOF-MS (Figure 2 and Figure 3). The high-resolution *N*-glycan fingerprints (migration time aligned and and peak height normalized electropherograms) from xCGE-LIF combined with the precise mass profiles generated by MALDI-TOF-MS allowed for fast and robust annotation also of isomeric *N*-glycan structures. Furthermore, normalization of *N*-glycan fingerprints to total peak height enabled relative quantification of individual *N*-glycan structures by xCGE-LIF. The glycans released by PNGase F from the insect-cell-produced recombinant SARS-CoV-2 spike glycoprotein are mainly *α*-1,6-core-fucosylated Man3F and G0F-Gn (3) structures (Figure 2A blue and Figure 3A). Moreover, Man2F, Man3, the hybrid-type structure G0-Gn (3), the complex-type structure G0F, and afucosylated oligomannose-type structures were detected. There is excellent agreement between xCGE-LIF and MALDI-TOF-MS measurements.

### *In-vitro* Glycoengineering of SARS-CoV-2 Spike Glycoprotein

Recombinant MGAT1ΔTM, MGAT2ΔTM and GalTΔTM were used in a one-pot glycoengineering reaction to convert the paucimannose structures to complex-type *N*-glycans. In scouting experiments it was found that after MGAT1ΔTM, MGAT2ΔTM and GalTΔTM addition at the start of the reaction, Man3F was converted to, at least in parts, to the hybrid-type structure G1F-Gn (3) missing the extension on the *α*1-6 mannosylated antenna catalysed by MGAT2. G1F-Gn (3) is not a natural substrate for MGAT2 and can, if at all, most likely only be processed at very low turnover rates. Thus, the reactions were carried out by adding the enzymes sequentially as detailed in M&M. In the first step, a GlcNAc residue is added from UDP-GlcNAc to the *α*-1,3-linked terminal mannose antenna of Man3F and Man3 by MGAT1ΔTM (Figure 2A and Figures 3A,B). After a reaction time of 12 h 24.7% of Man3 and 32% Man3F were converted to G0-Gn (3) and G0F-Gn (3), respectively. Scouting experiment showed that the conversion is typically irreversible and, thus, the incomplete processing is either due to low turnover or possible enzyme inactivation of MGAT1ΔTM. Another possibility is that the glycans are inaccessible for MGAT1ΔTM but can be released from the backbone by PNGase F. In the second step, UDP-GlcNAc and MGAT2ΔTM are added. After incubation for 12 h, the hybrid-type structures G0-Gn (3) and G0F-Gn (3) were converted to G0 and G0F with conversion rates of 100 and 85.2%, respectively (Figure 2B and Figure 3C). MGAT2ΔTM did not show any activity towards Man3 and Man3F. However, as mentioned before, this could be due the inaccessibility of these glycans. In the final step, the reaction was supplemented with UDP-galactose and GalTΔTM to add galactose to the terminal GlcNAc. At the end point of the reaction a conversion rate of 100% was achieved. The N-glycan fingerprint was now dominated by the galactosylated complex-type structure G2 along unprocessed Man3F (see Figure 2C and Figure 3D). Moreover, G0 was completely converted to G2 while the residual amount of the hybrid-type structure G0F-Gn (3) was also galactosylated to G1F-Gn (3). All oligomannose-type structures remained unaltered throughout the reaction. In general, the xCGE-LIF and the MALDI-TOF-MS data were in excellent agreement for all measurements.

## Discussion

Due to its scalability, eukaryotic protein processing and high productivity, the baculovirus-insect cell expression system is well-suited for the production of subunit vaccines (Felberbaum, 2015; Palomares et al., 2018). In addition to subunit vaccines against SARS-CoV-2 infections in development, there are currently three licensed vaccines, Flublok®, Cervarix® and Provenge® produced using this expression system with several more in clinical trials (Felberbaum, 2015; Palomares et al., 2018).

High immunogenicity of a recombinant insect-cell produced spike protein ectodomain variant, very similar to the one used here, has been confirmed in non-human primates [40]. Moreover, the spike protein is the antigen target of virtually all COVID-19 vaccines and advanced vaccine candidates (Krammer, 2020). At the time of writing this article, there was one licensed COVID-19 protein subunit vaccine (RBD-Dimer from Anhui Zhifei Longcom Biopharmaceutical, China) in China, while for two more candidates (Covovax from Novavax, United States; VAT00002 from Sanofi Pasteur and GSK, France/United Kingdom) emergency authorization was pending in the US and Europe (Yang et al., 2020; Dai and Gao, 2021; Shrotri et al., 2021). All three are recombinant SARS-CoV-2 spike protein variants produced using the baculovirus-insect cell expression system (Kyriakidis et al., 2021).

Glycoforms of recombinant proteins produced using baculovirus-insect cell expression systems are profoundly different from those produced using mammalian expression systems. An extensive review on the glycosylation processing of insect cells is given by Geisler et al. (2015). Typically, these proteins display mainly paucimannose and hybrid-type *N*-glycans with, at most, minor fractions of complex-type and oligomannose-type *N*-glycan (Geisler et al., 2015). Moreover, in comparison to *Spodoptera frugiperda* Sf9 cells, High Five® *Trichoplusia ni* cells can also produce core *α*-1,3-fucose-linked glycans (Palomares et al., 2018). The presence of the latter on biologics may cause hypersensitivity reactions when applied to patients with allergy and, thus, should be avoided, for instance by cell line engineering (Palmberger et al., 2014). According to the manufacturer’s information the recombinant SARS-CoV-2 spike protein used here was produced in the High Five® cell line. However, we excluded core *α*-1,3-fucose-linked glycans from the examination by using PNGase F that does not release this type of glycans from the protein backbone (Tretter et al., 1991).

For vaccine development, it has been proposed that immunogen candidates benefit from closely mimicking the macro- and microheterogeneity of the live virus glycosylation (Grant et al., 2020; Watanabe et al., 2020). This is as eliciting antibodies against shielded or non-native epitopes could cause an inefficient immune response. To overcome such obstacles, novel strategies utilizing distinct non-human glycans containing N-glycolylneuraminic acid or *α*,1-3 linked galactose residues, have been proposed to alleviate immune responses (Hütter et al., 2013; Galili, 2020; Schön et al., 2020; Chen, 2021). However, such approaches still need to be investigated in detail experimentally as, for example, both compounds are also suspected to cause allergenic reactions in humans.

To convert the glycoform from primarily paucimannose-type to typical mammalian complex-type *N*-glycans, the recombinant human glycosyltransferases, MGAT1ΔTM, MGAT2ΔTM and GalTΔTM, were effectively combined in a cell-free, one-pot glycosylation reaction. The gene expression of these glycosyltransferases in *E. coli* and the activity of the His-tag purified, soluble recombinant proteins in one-pot reactions using free glycans as substrates has been shown before (Fujiyama et al., 2001).

The site-specific glycan analysis of recombinant SARS-CoV-2 spike protein ectodomain expressed in human-derived cell line FreeStyle™ 293-F showed that of the 22 *N*-glycosylation sites only eight contained substantial fractions of oligomannose-type *N*-glycans (Watanabe et al., 2020). It is assumed that the occurrence of oligomannose-type fractions is caused by the steric inaccessibility of these glycans to the glycan processing enzymes in the Golgi, i.e., the occurrence of oligomannose-type *N*-glycans at distinct sites has shown to be independent of the producer cell line for the HIV viral glycoprotein gp120 (Pritchard et al., 2015). In accordance with the human cell-derived spike protein, our engineered spike protein abundantly exhibited complex-type G2F *N*-glycans. To a minor extend, a range of hybrid- and oligomannose-type *N*-glycans were also detected on the engineered spike protein. In contrast to the engineered spike protein, human cell-derived spike proteins also exhibit complex-type multi-antennary and sialylated structures (Watanabe et al., 2020). Taken together, a significant overlap of the glycoform has been generated. Whether the overlap is also site-specific remains to be investigated in future.

Over the past years, many efforts have been made to engineer insect cell lines to express complex-type *N*-glycans. A comprehensive summary of the attempts is given by Palomares et al. (2018). Briefly, complex-type *N*-glycans can be produced by the co-expression of glycosyltransferases or by generating transient insect cell lines. While the former generates an additional metabolic burden and affects growth properties, the stability of the latter has not been examined for commercial scale use. The advantage of *in-vitro* glycoengineering lies in its independence of producer cell lines as well as its flexibility towards the option to readily generate different glycoforms that are close to homogeneity. However, expensive nucleotides sugars are required as substrates and, thus, it is so far not feasible to apply *in-vitro* glycoengineering at larger scales (Mahour et al., 2018; Rexer et al., 2018; Rexer et al., 2020b).

## Conclusion

SARS-CoV-2 spike glycoprotein variants produced in a baculovirus-insect cell expression system were *in-vitro* glycoengineered using recombinant glycosyltransferases to mimic the glycoform observed on the human cell-derived protein. *In-vitro* glycoengineering reactions as conducted here, can be used to generate immunogen candidates for pre-clinical testing to investigate the role of glycosylation on the antigenicity and immunogenicity in animal models. In general, *in-vitro* glycoengineering approaches can virtually be used to tailor the glycoform of all prominent vaccine candidates such as activated and attenuated viruses and virus like particles. The application of the technology to larger scales depends on the bulk availability of sugar nucleotides at moderate costs.

## Data Availability

The original contributions presented in the study are included in the article/Supplementary Material, further inquiries can be directed to the corresponding author.

## Abbreviations

APTS, 8-Aminopyrene-1,3,6-trisulfonic acid; dH_2_O, Deionized water; GlcNAc, *N*-acetylglucosamine; HEPES, 4-(2-hydroxyethyl)-1-piperazineethanesulfonic acid; HILIC, Hydrophilic interaction liquid chromatography; His tag, Histidine tag; IMAC, Immobilized metal affinity chromatography; IPTG, Isopropyl β-D-1-thiogalactopyranoside; LC, Liquid chromatography; MALDI-TOF, Matrix-assisted laser desorption/ionization time-of-flight mass spectrometry; MS, Mass spectrometry; MnCl_2_, Mangan(II)-chlorid; MTU, Migration Time Units after alignment to internal standards; rpm, Rounds per min; SDS-PAGE, Sodium dodecyl sulfate polyacrylamide gel electrophoresis; SPE, Solid phase extraction; TPH, Total peak height; UDP-galactose, Uridine-diphosphate galactose; UDP-GlcNAc, Uridine diphosphate *N*-acetylglucosamine; xCGE-LIF, Multiplexed capillary gel electrophoresis with laser-induced fluorescence detection.
