# Supplementary material for: Cell-Free Glycoengineering of the Recombinant SARS-CoV-2 Spike Glycoprotein
Source: Front Bioeng Biotechnol. 2021 Aug 16;9:699025. doi: 10.3389/fbioe.2021.699025 (PMC8415157; doi:10.3389/fbioe.2021.699025)
Supplement: Supplementary file 1 [file DataSheet1.pdf]

Supplementary Material

1 Supplementary Data

1.1 Additional information on enzymes

For gene expression *E. coli* strains were typically grown to an OD_{600} of 0.6-0.8 in LB/TB media at 30°C followed by induction using 0.4 mM IPTG and a reduction of the cultivation temperature to 16°C. Plasmids with the gene insert were purchased from BioCat (Heidelberg, Germany).

Amino Acid Sequence:

MGAT1
TRPAPGRPPSVSALDGDPASLTREVIRLAQDAEVELERQRGLLQQIGDALSSQRGRVPTAAPP
AQPRVPVTPAPAVIPILVIACDRSTVRRCLDKLLHYRPSAELFPIIVSQDCGHEETAQAIASYG
SAVTHIRQPDLSSIAVPPDHRKFQGYYKIARHYRWALGQVFRQFRFPAAVVVEDDLEVAPDF
FEYFRATYPLLKADPSLWCVSAWNDNGKEQMVDASRPELLYRTDFFPGLGWLLLAELWAE
LEPKWPKAFWDDWMRRPEQRQGRACIRPEISRTMTFGRKGVSHGQFFDQHLKFIKLNQQFV
HFTQLDLSYLQREAYDRDFLARVYGAPQLQVEKVRTNDRKELGEVRVQYTGRDSFKAFAK
ALGVMDDLKSGVPRAGYRGIVTFQFRGRRVHLAPPLTWEGYDPSWN

MGAT2
RQRKNEALAPPLLDAEPARGAGGRGGDHPSVAVGIRRVSNVSAASLVPAVPQPEADNLTLR
YRSLVYQLNFDQTLRNVDKAGTWAPRELVLVVQVHNRPEYLRLLLDSLRKAQGIDNVLVIF
SHDFWSTEINQLIAGVNFCPVLQVFFPFSIQLYPNEFPGSDPRDCPRDLPKNAALKLGCINAEY
PDSFGHYREAKFSQTKHHWWWKLHFVWERVKILRDYAGLILFLEEDHYLAPDFYHVFKKM
WKLKQQECPECDVLSLGTYSASRSFYGMADKVDVKTWKSTEHNMGLALTRNAYQKLIECT
DTFCTYDDYNWDWTLQYLTVSCLPKFWKVLVPQIPRIFHAGDCGMHHKKTCRPSTQSAQIE
SLLNNNKQYMFPETLTISEKFTVVAISPPRKNGGWGDIRDHELCKSYRRLQ

Beta4GalT1
RDLSRLPQLVGVSTPLQGGSNSAAAIGQSSGELRTGGARPPPPLGASSQPRPGGDSSPVVDSG
PGPASNLTSVPVPHTTALSLPACPEESPLLVGPMLIEFNMPVDLELVAKQNPNVKMGGRYAP
RDCVSPHKVAIIIPFRNRQEHLKYWLYYLHPVLQRQQLDYGIYVINQAGDTIFNRAKLLNVG
FQEALKDYDYTCFVFSDVDLIPMNDHNAYRCFSQPRHISVAMDKFGFSLPYVQYFGGVSAL
SKQQFLTINGFPNNYWGWGGEDDDIFNRLVFRGMSISRPNAVVGRCRMIRHSRDKKNEPNP
QRFDRIAHTKETMLSDGLNSLTYQVLDVQRYPLYTQITVDIGTPS

1.2 Results

The recombinant enzymes were analyzed by SDS-PAGE after IMAC purification confirming production of all His-tagged variants (Supplementary Figure 1).

2 Supplementary Figures and Tables

2.1 Supplementary Figures

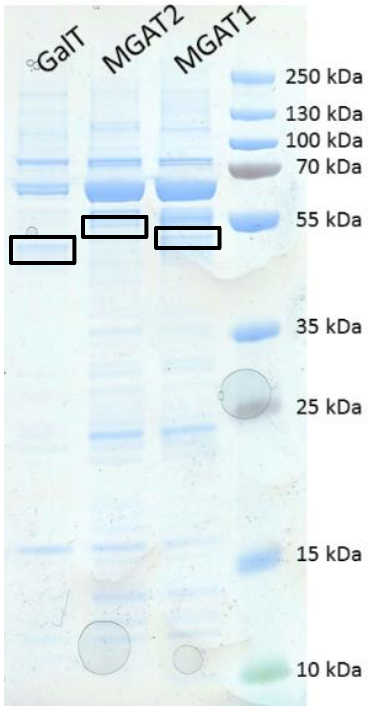

Supplementary Figure 1. SDS-PAGE (12% Bis-Tris) of 4 µg GalT, MGAT2 and MGAT1 each. Theoretical protein masses are: MGAT1ΔTM = 50.9 kDa, MGAT2ΔTM = 54.4 kDa , and GalTΔTM = 45.5 kDa .

2.2 Supplementary Tables

Supplementary Table 1. List of chemicals. Suppliers: AppliChem (Darmstadt, Germany), Applied Biosystems (Waltham, USA), Carl Roth (Karlsruhe, Germany), glyXera (Magdeburg, Germany), Merck (Darmstadt, Germany), Sigma Aldrich (St. Louis, USA) [now Merck], Thermphos International (Wittenberg, Germany), Thermo Scientific (Waltham, USA):

Chemical	Supplier	Product number	Purity
glyXprep16™ kit	glyXera	KIT001-16S	-
8-aminopyrene-1,3,6-trisulfonic acid (ATPS)	Sigma	09341	> 96%
Acetonitril	Thermo Scientific	A955	Optima™ “LC/MS grade”
BCA assay kit	Thermo Scientific	23227	-
Glycerol	Carl Roth	3783.1	>99.5%
HCl	Carl Roth	4025	37 %
HEPES	Carl Roth	9105.3	≥99.5 %
HiDi™-formamide	Applied Biosystems	4311320	-
Kanamycin sulfate	Merck	10106801001	-
LIZ™	Applied Biosystems	4322679	-
Imidazole	Carl Roth	3899.4	-
Isopropyl β-D-1-thiogalactopyranoside (IPTG)	AppliChem	A1008,0025	-
$MnCl_2$	Merck	1.05934.0100	-
NaCl	Carl Roth	P029.3	≥99 %
Trifluoroacetic acid	Merck	302031	>99%
Tris(hydroxymethyl)-aminomethan-buffer (TRIS)	AppliChem	A2264	> 99.9%)
Tryptone	Carl Roth	8952.2	-

UDP-GlcNAc	Carbosynth	MU07955	-
UDP-galactose	Carbosynth	MU06699	-
Yeast extract	Carl Roth	2363.2	

Supplementary Table 2. Genes, vectors and strains used for the synthesis of recombinant glycosyltransferases.

**Enzymes**	**Uniprot ID**	***E. coli* strain**	**Plasmid**
MGAT1	P26572	BL21(DE3)	pET-28a(+)
MGAT2	Q10469	SHuffle® T7 *lysY*	pET-28b(+)
GalT	P15291	BL21(DE3)	pET-28a(+)
